# Breaking the Triad: Immune Tolerance Induction Without Antigen Co-Presentation via Tim Agonist for the Treatment of Autoimmune Diseases

**DOI:** 10.3390/ijms26125531

**Published:** 2025-06-10

**Authors:** Basel Karzoun, Abdulraouf Ramadan, Saleh Allababidi, Anas M. Fathallah

**Affiliations:** LAPIX Therapeutics Inc., Cambridge, MA 02141, USA; basel.karzoun@lapixtherapeutics.com (B.K.); saleh.allababidi@lapixtherapeutics.com (S.A.)

**Keywords:** autoimmune, immune tolerance, antigen-agnostic, Tim3, Tim4

## Abstract

Autoimmune diseases such as multiple sclerosis (MS) are characterized by a loss of self-tolerance, driven by diminished regulatory T cell (Treg) function and elevated Th1/Th17 responses. Existing therapies broadly suppress the immune system without correcting this imbalance, often leading to adverse effects. LPX3, a novel small-molecule T cell immunoglobulin and mucin domain-containing 3 and 4 (Tim-3/4) receptor agonist, was developed to restore immune tolerance via Treg induction. In this study, LPX3 was formulated into a liposomal oral delivery system, enabling efficient uptake through the gastrointestinal tract and lymphatic targeting. In vitro and in vivo analyses confirmed LPX3’s ability to expand CD4^+^Foxp3^+^ Tregs in a dose-dependent manner. In a MOG-induced experimental autoimmune encephalomyelitis (EAE) mouse model of MS, both prophylactic and therapeutic oral administration of LPX3 significantly delayed disease onset, reduced symptom severity, and improved survival. Importantly, efficacy was achieved without antigen co-delivery, indicating an antigen-independent mechanism of immune modulation. LPX3 liposomes showed deep lymph node penetration and colocalization with immune cells, supporting its functional delivery to key immunological sites. These findings suggest LPX3 is a promising candidate for treating autoimmune diseases by re-establishing immune regulation through oral, antigen-agnostic tolerance induction.

## 1. Introduction

Autoimmune diseases, such as multiple sclerosis (MS), are chronic degenerative inflammatory conditions leading to immune-mediated damage to self. Although the precise pathogenesis of autoimmune disorders remains unclear, the loss of immune regulation is a consistent and critical common feature. This dysregulation is primarily driven by the downregulation of functional regulatory T cells (Tregs) and the upregulation of autoreactive Th1 and Th17 cells [1,2,3]. This imbalance results in sustained inflammation and contributes to the progressive nature of autoimmune diseases.

In MS, this imbalance results in immune-mediated myelin destruction, axonal damage and progressive neurodegeneration driven mainly by autoreactive CD4⁺ T cells, particularly Th1 and Th17 subsets, which recognize CNS antigens such as myelin basic protein (MBP) and myelin oligodendrocyte glycoprotein (MOG) among other myelin antigens [4]. Th1 cells secrete IFN-γ and TNF-α, activating macrophages and disrupting the blood–brain barrier (BBB), while Th17 cells produce IL-17 and IL-22, enhancing leukocyte infiltration and further compromising BBB integrity [3,5]. Environmental factors, particularly Epstein–Barr virus (EBV), have been strongly linked to MS risk. Nearly all MS patients show prior EBV infection, and elevated anti-EBV antibody titers support a causal association. Mechanisms include molecular mimicry [6,7], such as homology between EBNA-1 and the myelin-associated protein GlialCAM, leading to cross-reactive T and B cell responses that break immune tolerance [8,9]. These findings suggest that MS arises from a complex interplay between pathogenic Th1/Th17 responses, antigen mimicry, and environmental triggers like EBV, highlighting potential targets for therapeutic intervention.

Consequently, therapeutic strategies for autoimmune diseases have centered on broad immunosuppression rather than restoring immune tolerance. Current disease-modifying therapies (DMTs) include pleiotropic agents, immune cell-depleting therapies, anti-proliferative drugs, and agents that inhibit immune cells migration. While these approaches can reduce and/or modify disease activity and progression, they often carry significant risks, including infection, immune suppression, and long-term tolerability issues. Furthermore, none of these therapies directly address the fundamental loss of immune tolerance that drives disease progression.

Restoring immune tolerance in autoimmune diseases such as MS represents a promising strategy that could offer the potential of stable long-term remission to people afflicted by these diseases [10] without the adverse effects associated with broad immunosuppression. Antigen-specific tolerance induction for the treatment of autoimmune disorders in general and MS in particular has been evaluated clinically and non-clinically over the past 50 years [11,12]. These strategies typically rely on a triad of key components: (i) presentation of the antigen by a carrier, (ii) recognition of the antigen by immune cells, and (iii) the absence of co-stimulatory signals and/or the presence of an immunosuppressive agent, such as rapamycin [11]. Despite their potential, clinical translation has been limited by the difficulty of identifying relevant autoantigens, which are often variable or unknown.

Advancements in the understanding of immunoregulatory pathways, particularly those involving the T cell immunoglobulin and mucin domain-containing (Tim) family of receptors, have opened new avenues for autoimmune treatments via tolerance induction [13,14,15,16].

Tim-3 and Tim-4 are immunoregulatory receptors with complementary roles in modulating inflammation and maintaining immune tolerance. Tim-3, expressed on Th1, Th17, Tregs, NK cells, dendritic cells, and macrophages, mediates anti-inflammatory effects through ligands such as Galectin-9, phosphatidylserine (PS), HMGB1, and CEACAM1, activating inhibitory pathways (PI3K-AKT, MAPK-ERK, NF-κB) [17,18] that suppress effector T cell responses and enhance Treg function. In chronic inflammation, Tim-3 promotes immune exhaustion and is upregulated on APCs to limit antigen presentation. Tim-4, primarily on dendritic cells and macrophages, binds PS on apoptotic cells to promote their clearance and prevent autoantigen release, supporting peripheral tolerance [19]. Dysregulation of Tim-3 and Tim-4 is associated with autoimmune diseases such as multiple sclerosis, lupus, rheumatoid arthritis, and type 1 diabetes [20]. Targeting these checkpoints represents a potential therapeutic strategy to dampen autoimmunity and re-establish immune balance. In this study, we evaluated LPX3, a novel small-molecule Tim3/4 agonist designed to promote immune tolerance via Tim-mediated signaling in autoimmune diseases. Previously, we have demonstrated the use of LPX3 to induce highly functional CD4^+^Foxp3^+^CTLA4^+^ICOS^lo^ tolerogenic regulatory T cells (T-regs) for antigen-specific tolerance to AAV9 [21]. Here, we extend these findings by evaluating its potential to induce both antigen-dependent and antigen-independent tolerance, with the aim of developing an antigen-agnostic therapeutic strategy for autoimmune disease.

We first incorporated LPX3 into a liposomal formulation suitable for oral delivery, a route that not only confers unique immunological advantages [22,23] but also improves patient adherence and convenience [24]. We then characterized the pharmacological properties of the optimized LPX3 formulation in vitro and in vivo before assessing its efficacy in a MOG-induced experimental autoimmune encephalomyelitis (EAE) mouse model of MS.

## 2. Results

### 2.1. Optimization of DilC18(5) Dye Incorporation in Liposomes

Fluorescence intensity measurements of DilC18(5) in PBS across 0.1–2 µM confirmed its weak signal in aqueous media. In contrast, when incorporated into liposomes, relative fluorescence intensity (RFI) increased more than 20-fold between 0.1 and 0.5 µM, with further enhancement at 1 µM. At 2 µM, a decrease in RFI was observed, possibly due to self-quenching or suboptimal incorporation. These results indicate that 0.1 µM is sufficient for sensitive detection in liposomal formulations, eliminating the need for a purification step.

### 2.2. Liposomal Formulations and Lymphatic Uptake

#### 2.2.1. Relative Fluorescence Intensity by Lipid Type

**Figure 1:**
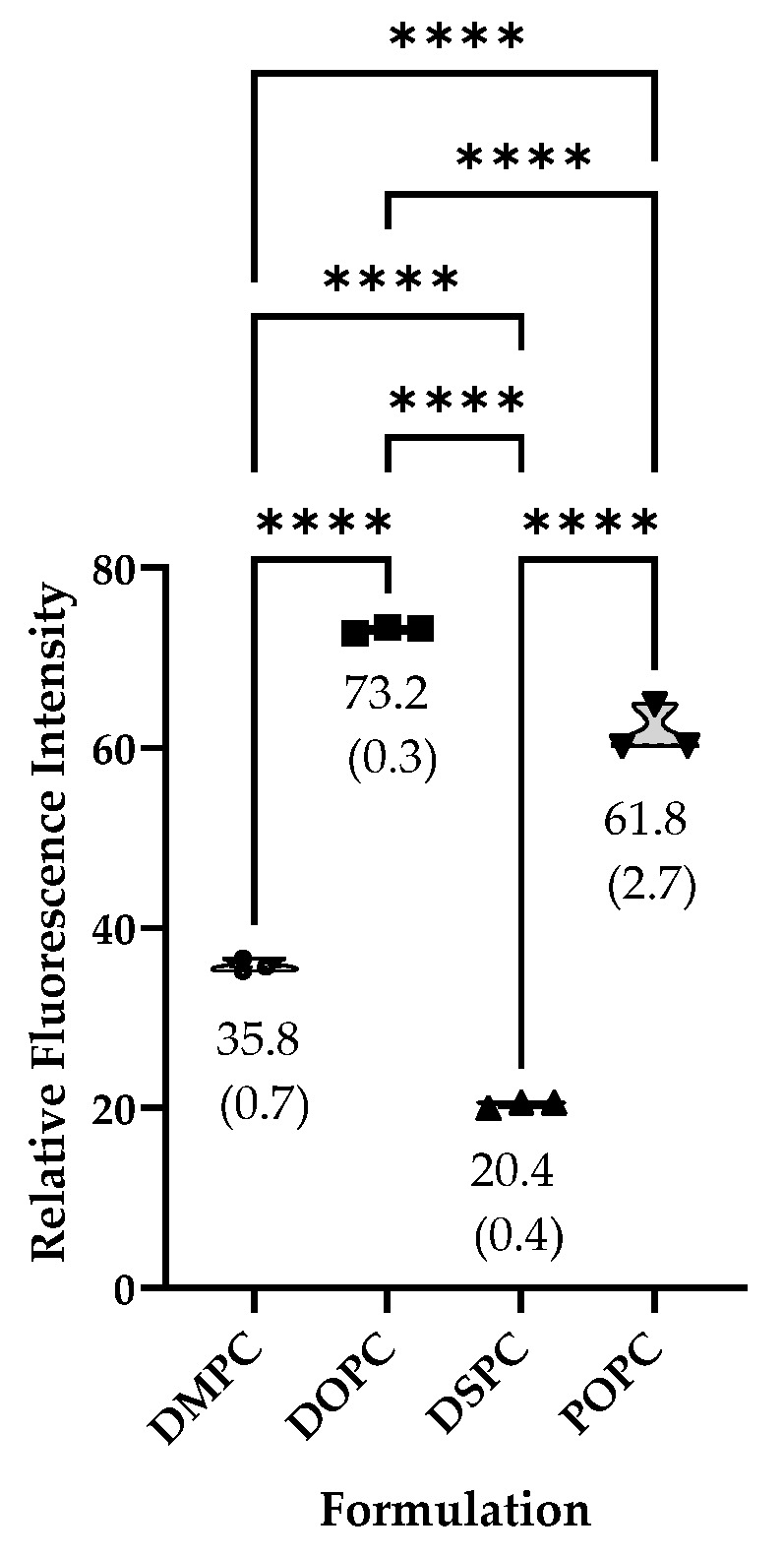
The relative fluorescence intensity of DilC18(5) dye is higher in liposomes composed of unsaturated phospholipids (DOPC and POPC) vs. those composed of saturated lipids (DMPC and DSPC). **** *p* < 0.0001, ordinary one-way ANOVA with multiple comparisons.

The RFI of liposomes composed of unsaturated phospholipids (DOPC and POPC) was higher than that of saturated lipids (DMPC and DSPC) (73.2 and 61.8) vs. (35.8 and 20.4), respectively, suggesting differences in dye Incorporation or liposomal properties based on lipid saturation (Figure 1).

#### 2.2.2. Fluorescent Immunohistochemistry and Free DilC18(5) Uptake

**Figure 2:**
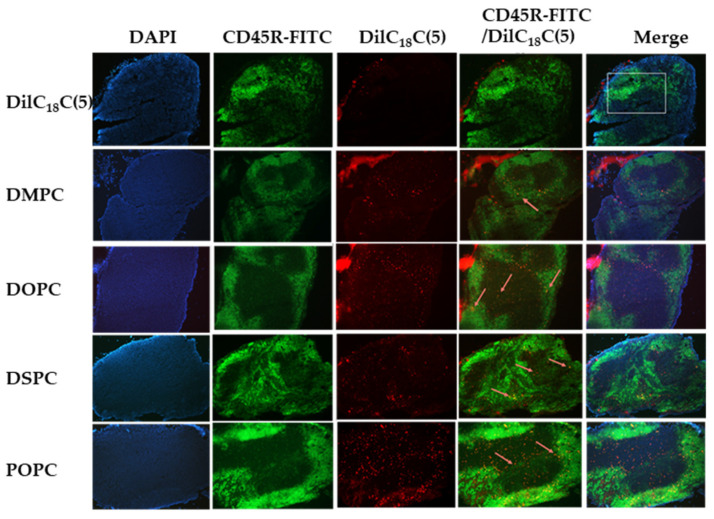
Representative images of B cell (CD45R-FITC) fluorescence immunostaining and DilC18(5) liposome colocalization with B cells 60 min after oral gavage of either free dye or different liposomal formulations in mice. The DilC18(5) panel shows no fluorescence of the free DilC18(5) dye in the core of the lymph node section and no colocalization with B cells. In contrast, all liposomal formulation containing DilC18(5) showed penetration of the lymph node and colocalization with B cells (orange arrows in the CD45R-FTIC/DilC18(5) panels). Blue represent DAPI staining of cell nuclei, Green represents B-cells stained with FITC, Red represent DilC18(5) labeled liposomes in lymph nodes, Orange represents colocalization of Green and Red (B-cells with liposomes). All images acquired at 10× magnification.

**Figure 3:**
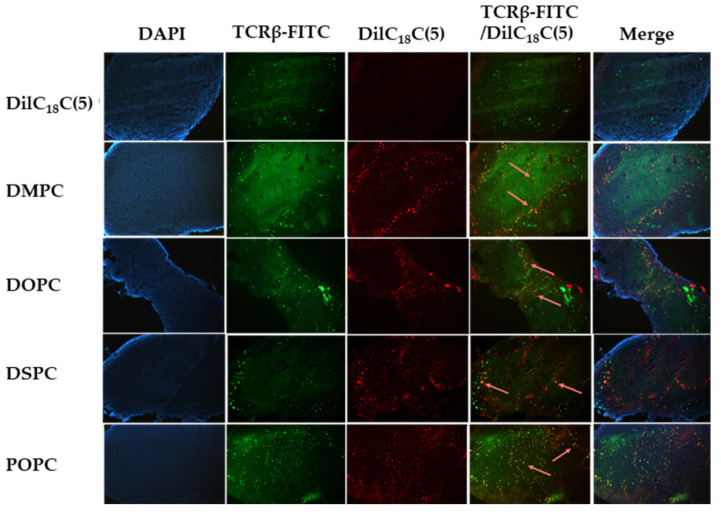
Representative images of T cell (TCRβ-FITC) fluorescence immunostaining and DilC18(5) liposome colocalization with T cells 60 min after oral gavage of either free dye or different liposomal formulations in mice. The DilC18(5) panel shows no fluorescence of the free DilC18(5) dye in the core of the lymph node section and no colocalization with T cells. In contrast, all liposomal formulations containing DilC18(5) showed penetration of the lymph node and colocalization with T cells (orange arrows in the TCRβ-FTIC/DilC18(5) panels). Blue represents DAPI staining of cell nuclei, Green represents T-cells stained with FITC, Red represents DilC18(5) labeled liposomes in lymph nodes, Orange represents colocalization of Green and Red (T-cells with liposomes). All images acquired at 10× magnification.

All lymph node sections stained positive for B cells (CD45R-FITC) and T cells (TCRβ-FITC), with both mature (bright) and immature (dim) cell populations observed. B cell clustering was evident in cortical regions, and T cells were generally dispersed throughout the paracortex. Minimal to no DilC18(5) signal was detected in mesenteric lymph nodes 60 min post-oral administration of free dye, with no colocalization with either B or T cells (Figure 2 and Figure 3, DilC18(5) panel).

#### 2.2.3. Liposome Uptake and Immune Cell Colocalization by Formulation

The uptake of each formulation of LPX3 liposomes (160 µM, 200 µL oral dose) was evaluated at 60 min post-gavage. Liposomes composed of DMPC primarily localized with mature B cells (bright CD45R signal) (Figure 2) and clustered T cells (Figure 3). In contrast, DOPC showed deeper lymph node penetration and colocalization with both mature and immature B cells (Figure 2) and T cells (Figure 3). DSPC exhibited similar properties to DOPC (Figure 2 and Figure 3), with broad distribution and colocalization with both B and T cells, including diffuse red signal indicating broader dispersion. POPC also demonstrated deep lymph node penetration and colocalization with both mature and immature immune cells (Figure 2 and Figure 3).

#### 2.2.4. Quantitative Colocalization Analysis

##### B Cell Colocalization

**Figure 4:**
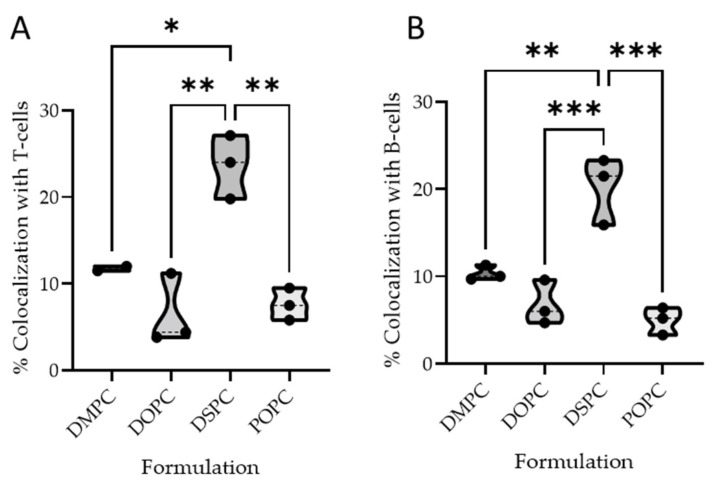
Percent colocalization of different liposomal formulation with B cells (**A**) and T cells (**B**) in mesenteric lymph node 60 min after oral administration in mice. DSPC liposomes outperformed other liposomal formulations (ordinary one-way ANOVA with multiple comparisons, * *p* < 0.05, ** *p* < 0.01, *** *p* < 0.001).

The B cell colocalization was 20.2% (SD = 3.9) for DSPC, 10.3% (SD = 0.8) for DMPC, 6.8% (SD = 2.5) for DOPC, and 5.0% (SD = 1.6) for POPC. Statistical analysis confirmed that DSPC significantly outperformed all other formulations for B cell targeting (Figure 4A).

##### T Cell Colocalization

Similar to B cell colocalization, DSPC significantly outperformed DOPC and POPC in T cell colocalization. Specifically, DSPC showed 23.6% (SD = 3.7), DMPC 26.9% (SD = 26.2, high variance), DOPC 6.5% (SD = 4.1), and POPC 7.6% (SD = 1.8) (Figure 4B).

### 2.3. Pharmacology of LPX3 Liposomes

#### 2.3.1. In Vitro Pharmacology

**Figure 5:**
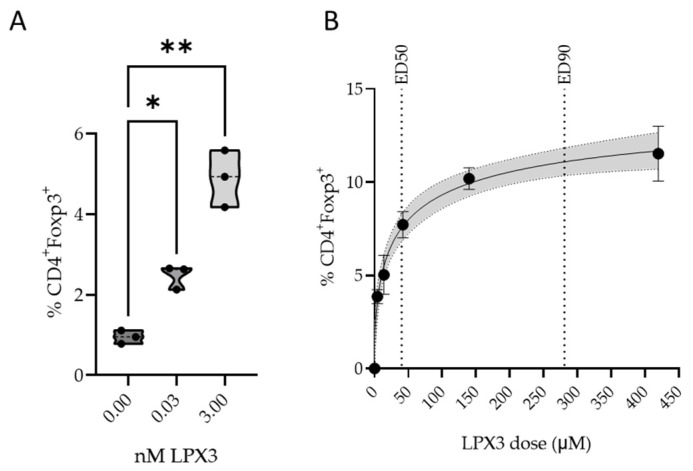
Changes in percent CD4^+^Foxp3^+^ T cells (T-regs) in response to LPX3 liposomes in vitro (**A**) and after oral dosing in vivo, gray shaded area represents the confidence interval about the fitted dose-response (**B**). Ordinary one-way ANOVA with multiple comparisons; * *p* < 0.05, ** *p* < 0.01 was used in (**A**); EC50 and EC90 were obtained by model fitting of data to a 4-parameter hill function for (**B**).

LPX3 formulated in DSPC liposomes resulted in a statistically significant and dose-dependent increase in CD4^+^Foxp3^+^ T cells (Tregs) in culture compared to untreated splenocytes incubated under the same conditions. Tregs increased from a mean (SD) of 0.94% (0.16) to 2.4% (0.269) and 4.8 T (0.7) in splenocytes treated with 0.03 and 3 nM LPX3, respectively (Figure 5A).

#### 2.3.2. In Vivo Pharmacology

Oral administration of LPX3 formulated in DSPC liposomes induced a dose-dependent increase in CD4^+^FoxP3^+^ T cells in mice. Tregs increased from a mean of 3.87% (SD = 0.37) at 4.2 µM to 11.53% (SD = 1.47) at 140 µM. Model fitting estimated an ED_50_ of 43.1 µM and an ED_90_ of 281.0 µM. The model captured the observed data well, with a maximum estimated effect (Emax) of 12.1% CD4^+^FoxP3^+^ cells. An oral dose of 42 µM was used for all efficacy studies in mice (Figure 5B).

### 2.4. Effect of LPX3 Liposomes in MOG_35–55_ Experimental Autoimmune Encephalomyelitis (EAE) Model

#### 2.4.1. Prophylactic Effects of LPX3 and LPX3/MOG_35–55_ in the EAE Model

**Figure 6:**
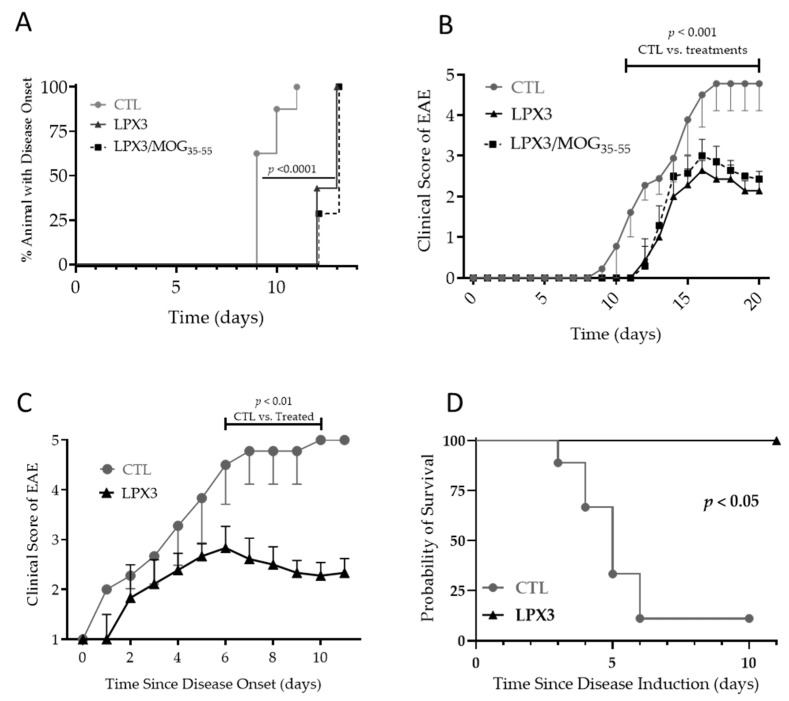
Effects of LPX3 liposomes in a MOG_35–55_ experimental autoimmune encephalomyelitis (EAE) model. Mice treated with LPX3 liposomes with or without MOG_35–55_ showed a statistically significant delay in disease onset compared to untreated mice (**A**). Disease progression and clinical scores were the same between LPX3 liposomes with or without MOG_35–55_, suggesting that antigen co-presentation is not required to induce the observed clinical benefit (**B**). In a therapeutic context (i.e., treatment started after disease onset), mice treated with LPX3 liposomes showed better disease control compared to untreated mice (**C**). All (9/9) mice treated with LPX3 survived the study vs. 1/9 mice in the untreated group (**D**).

Disease onset was delayed in both the LPX3 and LPX-3/MOG_35–55_ groups compared to the control group. In the control group, onset started on day 9 after immunization, whereas in the treatment groups, onset started on day 12. All animals in the treatment groups exhibited symptoms by day 13, compared to day 11 in the control group. Time-to-event analysis showed a statistically significant delay in disease onset for both treatments, with no difference between LPX3 and LPX3/MOG_35–55_ (Figure 6A).

After disease onset, both the LPX3 and LPX3/MOG_35–55_ groups exhibited milder symptoms compared to the control group. Peak severity in the control group occurred on day 17, with a mean clinical score of 4.77 (SD = 0.67). In contrast, the LPX3 and LPX3/MOG_35–55_ groups peaked on day 17 with mean scores of 2.6 (SD = 0.23) and 2.9 (SD = 0.41), respectively. Both treatment groups showed a recovery phase, with mean scores dropping to 2.1 (SD = 0.23) for LPX3 and 2.4 (SD = 0.23) for LPX3/MOG_35–55_ by the end of the study. In contrast, no recovery phase was observed in the control group, where most animals died (Figure 6B).

#### 2.4.2. Therapeutic Effects of LPX3 in the EAE Model

##### Disease Progression and Clinical Scores

In the therapeutic paradigm, treatment was initiated at clinical score of 1. Treatment with LPX3 resulted in a one-day delay in disease progression, with milder symptoms compared to untreated controls. Peak mean score of the untreated group was 4.77 (SD = 0.67), vs. a mean score of 2.80 (SD = 0.43) for the LPX3-treated group. Symptoms in the treatment group subsided after day 6, reaching a mean score of 2.33 (SD = 0.29) by day 10. The control group did not exhibit remission due to high mortality (Figure 6C).

##### Overall Survival in Treated vs. Untreated Mice

Survival analysis showed a stark contrast between the treatment groups. In the control group, 8 out of 9 animals either died or were euthanized, whereas no deaths occurred in the LPX3-treated group. This study featured an unusually aggressive EAE model, as typical mortality rates in EAE models are generally less than 30% (Figure 6D).

#### 2.4.3. Ex Vivo Analysis of CD4^+^Foxp3^+^ T Cells in Treated vs. Untreated Mice

**Figure 7:**
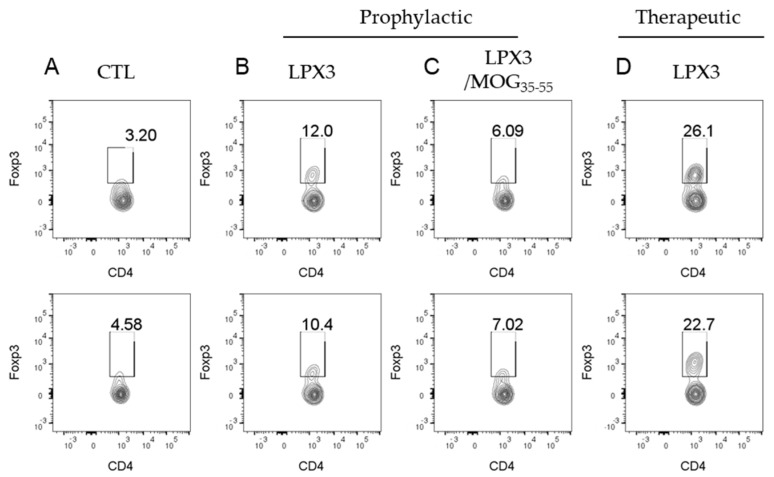
Flow cytometry plots showing CD4⁺Foxp3⁺ (Treg) expression in EAE mice at the end of this study for control untreated mice (**A**), mice treated prophylactically with LPX3 (**B**) and LPX/MOG_35–55_ (**C**), or mice treated after disease onset (therapeutically) with LPX3 (**D**).

Flow cytometry analysis of splenocytes revealed a significant increase in CD4^+^Foxp3^+^ T cells in all treatment groups. In the prophylactic groups, LPX3 (Figure 7B) and LPX-3/MOG_35–55_ (Figure 7C) showed an average increase of 187.8% and 68.3%, respectively, compared to the control (Figure 7A) (11.2% and 6.55% vs. 3.89%). In the therapeutic group (Figure 7D), LPX3-treated mice showed an average increase of 527.2% in CD4^+^Foxp3^+^ T cells compared to the untreated control (24.4% vs. 3.89%).

## 3. Discussion

Despite its immense potential, the clinical and commercial application of immune tolerance induction for the treatment of autoimmune diseases has been hindered by the need for antigen co-presentation. This requirement necessitates a prior knowledge of the relevant pathogenic antigen(s) and their presentation to the immune system alongside an immunomodulatory agent [11,25]; however, in the context of autoimmune diseases, this can be particularly challenging, as the specific autoantigens are often variable or entirely unknown.

**Figure 8:**
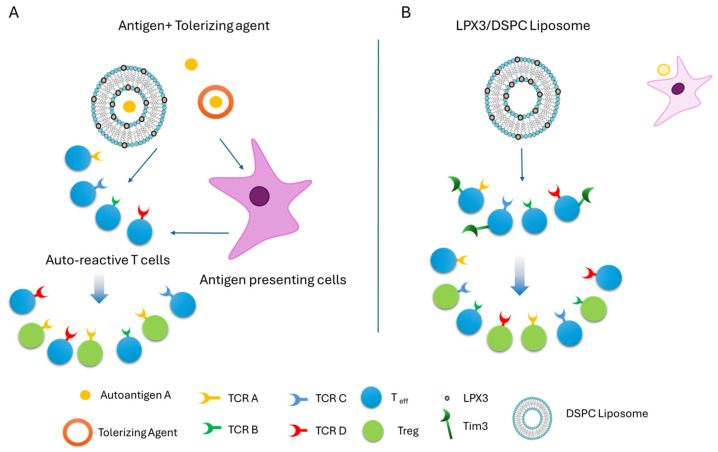
Illustration of the triad of antigen-specific tolerance induction. (**A**) The autoantigen “A” is presented using a carrier or tolerizing agent. Only T cells expressing TCR “A”—specific for this autoantigen “A”—will be engaged in the process, leading to the generation of antigen-specific regulatory T cells (Tregs). (**B**) In contrast, the Tim-3 agonist LPX3 activates the natural tolerance pathway without requiring antigen presentation or engagement of TCR. This induces antigen-agnostic tolerance, which is especially beneficial in conditions involving multiple autoantigens or epitope spreading.

Previously, we demonstrated that LPX3 induces antigen-specific tolerance to AAV9 without disrupting innate immune responses through the agonism of Tim family receptors [10], which relies on the triad of tolerance induction, i.e., (i) presentation of the antigen by a carrier, (ii) recognition of the antigen by immune cells, and (iii) the absence of co-stimulatory signals and/or presence of an immunomodulatory compound (Figure 8A).

In the present study, we investigated the use of LPX3 to induce antigen-agnostic immune tolerance in the context of autoimmune disease. Because both the autoantigen and autoreactive T cells are already present together in these conditions, we hypothesized that targeting the Tim pathway—a well-characterized natural tolerogenic mechanism of the body [7,8,9]—would expand regulatory T cell (Treg) populations, thereby promoting immune tolerance and mitigating disease progression (Figure 8B).

Tim-3 and Tim-4 are immunoregulatory receptors critical to maintaining immune tolerance naturally [26,27]. Tim-3, in particular, is recognized as a checkpoint for T cell activation and for limiting pathological inflammation in autoimmune settings [28,29,30]. Supporting this, Tim-3 knockout mice exhibit exacerbated EAE compared to wild-type controls [14], underscoring Tim-3 as a compelling therapeutic target for LPX3. The ability of LPX3 to induce immune tolerance without requiring antigen co-presentation represents a significant advance over traditional antigen-specific strategies. By circumventing the need for defined autoantigens—heterogeneous in diseases like MS or undefined in other autoimmune diseases—LPX3 introduces a novel, broadly applicable mechanism of immune modulation.

Beyond its direct effects on Tregs, LPX3’s mechanism may also involve broader immune modulation, given that Tim-3 and Tim-4 are expressed on a variety of immune cells, including antigen-presenting cells (APCs). It has been shown that bone marrow-derived dendritic cells (BMDCs) treated ex vivo with a Tim-3/4 ligand (OPLS) can induce Tregs in recipient mice [31], supporting the idea of indirect Treg activation. In the context of autoimmune diseases such as MS, ongoing tissue damage and epitope spreading release self-antigens into circulation, which may further reduce the necessity of incorporating specific autoantigens into the liposomal formulation.

Oral delivery offers multiple advantages, including convenience, improved patient compliance, and systemic accessibility. Our data demonstrates that liposomes embedding LPX3 within their lipid bilayer are localized to lymphoid tissues where T and B cell interactions occur [32,33]. Following oral administration, LPX3 traffics through the gastrointestinal tract and accumulates in mesenteric lymph nodes, with robust colocalization observed in both B and T cell populations. This deep lymph node penetration enables effective engagement of Tim receptors on immune cells. Notably, oral LPX3 administration induced a dose-dependent systemic expansion of Tregs in mice.

This systemic Treg induction translated into meaningful clinical benefits in the EAE model. In prophylactic administration, LPX3 delayed disease onset and reduced clinical severity, irrespective of co-presentation with the MOG_35–55_ peptide. This distinguishes LPX3 from antigen-specific immune tolerance approaches and enabled its evaluation in a therapeutic paradigm. When administered after clinical onset, LPX3 treatment delayed disease progression and significantly reduced symptom severity compared to controls, with a concurrent increase in circulating Tregs.

Our results highlight the promising therapeutic potential of LPX3 in modulating immune responses and restoring immune tolerance in a murine model of multiple sclerosis (MS). LPX3 treatment attenuated disease symptoms and delayed onset in experimental autoimmune encephalomyelitis (EAE), a well-established model of MS pathology. Furthermore, LPX3’s liposomal formulation, optimized for oral administration, enhances its potential as a practical and effective therapeutic for autoimmune diseases.

An important consideration in interpreting LPX3’s mechanism involves the dual functionality of Tim receptors. For instance, Tim-3 on NK cells can act as either an activator or inhibitor depending on the immune context. During acute immune responses, Tim-3 enhances NK cytotoxicity and IFN-γ production [34], while in chronic inflammation or cancer, its engagement—particularly by Galectin-9—suppresses NK activity and cytokine production, contributing to immune exhaustion [35]. Notably, Tim-3 is upregulated in cancer and chronic viral infections, impairing T cell effector functions. In contrast, in autoimmune disease, Tim-3 is typically downregulated, reducing Treg expression and function. These distinctions support the rationale for selectively engaging Tim receptors in autoimmunity without broadly dampening immune defense [36,37].

Previously, we demonstrated that engaging Tim with our agonists induces immune tolerance without broadly suppressing innate immunity, particularly sparing transmembrane TLRs (TLR2, TLR4) and partially affecting endosomal TLR9 [21]. Therefore, we hypothesize that LPX3 does not fully suppress innate responses.

While this study focused on MS, the implications extend to other autoimmune diseases due to LPX3’s antigen-agnostic mechanism and ability to re-establish immune tolerance. Additionally, its oral formulation offers a favorable alternative to injectable or infusible biologics, supporting its use in long-term disease management.

Further work will focus on optimizing LPX3’s liposomal formulation to improve bioavailability and tissue-specific delivery. Additionally, studies investigating the long-term immunologic effects of oral LPX3 treatment will be essential to ensure safety and efficacy in chronic use.

## 4. Materials and Methods

### 4.1. Materials

Lipids including DMPC, DSPC, DOPC, POPC (Avanti Polar Lipids, Alabaster, AL, USA), DilC18(5) dye, chloroform, PBS, OCT compound, PFA, DMSO (Thermo-Fisher, Waltham, MA, USA), FITC-CD45R and FITC-TCR-β antibodies (Invitrogen, Waltham, MA, USA), and DAPI (Vector Laboratories, Newark, CA, USA) were used. LPX3 was obtained from (LAPIX Therapeutics, Cambridge, MA, USA). Key equipment included a rotavapor (Buchi, Flawil, Switzerland), liposome extruder (Avanti Polar Lipids, Alabaster, AL, USA), Branson Ultrasonics bath (Emerson Electronics, Brookfield, CT, USA), and a Nikon E-800 epifluorescence microscope (Nikon Corporation, Tokyo, Japan) image analysis was done with ImageJ software (Version 1.53e, National Institutes of Health, Bethesda, MD, USA).

### 4.2. Animal Housing and Handling

C57BL/6 female mice (aged 7–8 weeks) were purchased from Charles River Laboratories (Wilmington, MA, USA) and maintained under specific pathogen-free conditions at the LAPIX Therapeutics Inc. (Cambridge, MA, USA) animal facility. Mice were housed on a 12 h light/dark cycle with unrestricted access to food and water. All animal studies at LAPIX Therapeutics Inc. are conducted under IACUC number EB17-029-303 administered by CRADL, Charles River.

### 4.3. DilC18(5) Dye Screening

DMPC liposomes were prepared by dissolving DMPC in chloroform and forming a thin lipid film via rotavapor. The film was hydrated with PBS containing DilC18(5) dye (0.1–2 µM). Fluorescent intensity was measured in a 96-well plate at Ex/Em 650 nm/670 nm to identify optimal dye concentration.

### 4.4. Liposomal Formulation Preparation for Colocalization Studies

Four liposomal formulations were prepared using a 3.75:96.25 LPX3/lipid molar ratio (DMPC, DSPC, DOPC, or POPC). LPX3 was dissolved in DMSO, evaporated in a rotavapor, and combined with lipid solutions. After forming a thin film, the formulations were hydrated with PBS and DilC18(5) dye (0.1 µM) to create a 160 µM dispersion. Fluorescent intensities were measured at Ex/Em 650 nm/670 nm.

### 4.5. Liposomal Formulation for MOG-EAE Studies

Two formulations were tested: LPX3:DSPC (3.75:96.25) and LPX3/MOG_35–55_ (LPX3 combined with MOG_35–55_ peptide). The liposome film was prepared as above. For LPX3-MOG_35–55_, 10 µg/mL of MOG_35–55_ was added to the hydration buffer.

### 4.6. Lymphatic Uptake Quantification

Mice (n = 3/group) were dosed with 200 µL of 160 µM fluorescent liposome formulation via oral gavage. The control group received PBS with 0.1 µM DilC18(5). After 60 min, mesenteric lymph nodes were harvested, preserved in OCT, sectioned, and analyzed for liposome–immune cell colocalization.

Immunofluorescence Staining for T and B Cells: Frozen lymph node sections were fixed in 2% PFA, stained overnight with FITC-CD45R (B cells) or FITC-TCR-β (T cells) antibodies, and mounted with DAPI.

#### 4.6.1. Fluorescence Imaging

Sections were imaged using a Nikon epifluorescence microscope with Cy5 (Ex/Em 650 nm/670 nm), FITC (Ex/Em 494 nm/518 nm), and DAPI (Ex/Em 358 nm/461 nm) channels.

#### 4.6.2. Colocalization Analysis

ImageJ software was used for colocalization analysis. The percent area of colocalization was calculated by merging images and thresholding the yellow spectrum, representing the colocalization of immune cells (green) and liposomes (red). The results were normalized by the RFI of each liposome to account for formulation-related differences in RFI and compared to control group data. Since our interest was to determine the effectiveness of liposome/immune cell interaction, we needed to account for the presence of the immune cells in the particular section. This was performed by normalizing the corrected mean FI of the colocalized area to the mean FI of immune cells using the following formula to obtain a colocalization value that would allow us to compare and contrast the results:

$$\%Colocalization=\frac{corrected\;mean\;FI\;for\;each\;section}{mean\;FI\;for\;immune\;cells\;in\;each\;section\;}\times 100$$

### 4.7. In Vivo Pharmacology

Female B57BL/6 mice (n = 3 per group) were housed under standard conditions and received a single oral dose of LPX3:DSPC 4.2, 14, 42, or 140 µM of LPX3 (100 µL volume). Five days post-dosing, spleens were collected for ex vivo analysis. Splenocytes were stained and analyzed via flow cytometry to quantify FoxP3^+^/CD4^+^ regulatory T cells. Dose–response data were modeled using four-parameter logistic regression in R (version 4.5.0) (drc package version 3.0-1) to estimate ED_50_ and ED_90_ values.

### 4.8. EAE Study

Experimental Autoimmune Encephalomyelitis (EAE) Induction: Eight- to nine-week-old female mice were immunized with 100 µg MOG (Bio-Techne, Minneapolis, MN, USA) in complete Freund’s adjuvant (CFA, BD Difco, Franklin Lakes, NJ, USA) containing 500 µg Mycobacterium tuberculosis H37. Pertussis toxin (200 ng, List Biological Laboratories, CA, USA) was administered intravenously on days 0 and 2 post-immunization. Disease severity was scored on a 5-point scale as follows: (0) No clinical signs. (1) Tail paralysis. (2) Hind limb weakness, loss of righting reflex. OR Mouse appears to be at a score 0.0, but there are obvious signs of head tilting when walking. The balance is poor. (2.5) One hind limb paralyzed. (3) Two hind limbs paralyzed. (3.5) Two hind limbs paralyzed and one front limb paralyzed. (4) Quadriplegia. (5) Found dead in cage or euthanized due to severe morbidity as described in [7]. Since no sex differences are noted in MOG_35–55_ induced EAE in C57BL/6 mice [38], the authors opted to used female mice only for ease of handling and housing. 

#### 4.8.1. Group Assignment

##### Prophylactic

Mice were treated with LPX3 liposomes (100 µL of 42 µM) or LPX3/MOG_35–55_ liposomes (LPX3 co-formulated with 1 µg MOG_35–55_) orally starting 4 days post-immunization.

##### Therapeutic

Treatment started after the first clinical symptoms were observed. Mice received LPX3 liposomes (100 µL of 42 µM) orally until this study’s end.

##### Survival Analysis

Mice were monitored for clinical symptoms. Animals with a clinical score of 5 or those found dead were considered events for survival analysis.

#### 4.8.2. Ex Vivo Splenocyte Isolation and Flow Cytometry Analysis

At study termination, spleens were aseptically harvested and processed into single-cell suspensions by mechanical dissociation over a 70 µm cell strainer. Cells were resuspended in DMEM supplemented with 10% FBS, and red blood cells were lysed using RBC lysis buffer (Thermo Fisher Scientific, Waltham, MA, USA) for 5 min at room temperature. Following lysis, cells were washed and resuspended in complete DMEM. For flow cytometry analysis, splenocytes were first blocked with anti-CD16/CD32 (clone 93, Thermo Fisher Scientific, Waltham, MA, USA) and then stained with fluorochrome-conjugated antibodies against surface markers CD3 and CD4 (clone GK1.5, Thermo Fisher Scientific, Waltham, MA, USA), as well as intracellular Foxp3 (Thermo Fisher Scientific, Waltham, MA, USA). Live/dead cell discrimination was performed using Fixable Viability Dye eFluor 780. Intracellular staining was conducted following fixation and permeabilization using the Transcription Factor Staining Buffer Set (Thermo Fisher Scientific, Waltham, MA, USA). Flow cytometry was performed on an Attune NxT Flow Cytometer (Thermo Fisher Scientific, Waltham, MA, USA), and data were analyzed using FlowJo v10 software (TreeStar, Ashland, OR, USA) to assess the frequency of CD4⁺Foxp3⁺ regulatory T cells (Tregs).

## Data Availability

The data presented in this study are available on request from the corresponding author. (The data is not publicly available due to potential IP and confidentiality restrictions).

## Abbreviations

The following abbreviations are used in this manuscript:

DMPC	1,2-dimyristoyl-sn-glycero-3-phophocholine
DOPC	1,2-dioleoyl-sn-glycero-3-phophocholine
DSPC	1,2-distearoyl-sn-glycero-3-phophocholine
POPC	1-palmitoyl-2oleoyl-sn-glycer-3-phosphocholine
MOG	Myelin oligodendrocyte glycoprotein
